# Aging unconventionally: γδ T cells, iNKT cells, and MAIT cells in aging

**DOI:** 10.1016/j.smim.2023.101816

**Published:** 2023-09

**Authors:** Ayako Kurioka, Paul Klenerman

**Affiliations:** a Nuffield Department of Medicine, University of Oxford, Oxford, UK; b Translational Gastroenterology Unit, University of Oxford, Oxford, UK

**Keywords:** Gamma delta T cells, MAIT cells, INKT cells, Aging, Cancer, Inflammaging

## Abstract

Unconventional T cells include γδ T cells, invariant Natural Killer T cells (iNKT) cells and Mucosal Associated Invariant T (MAIT) cells, which are distinguished from conventional T cells by their recognition of non-peptide ligands presented by non-polymorphic antigen presenting molecules and rapid effector functions that are pre-programmed during their development. Here we review current knowledge of the effect of age on unconventional T cells, from early life to old age, in both mice and humans. We then discuss the role of unconventional T cells in age-associated diseases and infections, highlighting the similarities between members of the unconventional T cell family in the context of aging.

## 1 Introduction

Aging is associated with profound changes in the immune system that result in increased vulnerability to infectious diseases, cancer and autoimmune diseases, as well as reduced vaccine efficacy. The increased incidences of diseases observed in older individuals is thought to be due to the progressive dysfunction of the immune system. Immunosenescence is the gradual decline in the acquired immune response to foreign pathogens, most significantly due reduced numbers of naïve T cells [1], accumulation of memory T cells [2], reduced B cell numbers and function [3], increased immunosuppression by regulatory T cells (T_regs_) [4], as well as dysfunction in the innate immune system in granulocytes, monocytes/macrophages, and Natural Killer (NK) cells [5]. A striking feature of immunosenescence is the excessive production of proinflammatory cytokines that increases with age, including Interleukin 1β (IL-1β), IL-6, IL-8, tumour necrosis factor α (TNFα), IL-18, as well as chemokines such as Chemokine CC motif ligand 5 (CCL5) and monocyte chemoattractant protein (MCP-1), and this aging-associated proinflammatory state is called inflammaging [6], [7]. Inflammaging is further accelerated by age-induced dysbiosis, which is the changing composition of component microorganisms of commensal microbiota that occurs with advancing age, associated with changes in the gut barrier immune cells and increased gut permeability [8]. These complex age-related changes are closely associated and pave the way to accelerated aging and poor outcomes of several illnesses, most recently highlighted by the coronavirus disease 2019 (COVID-19) pandemic [9].

Age-associated changes in conventional T cells, which express an αβ T cell receptor (TCR) and recognise peptides presented by polymorphic major histocompatibility complex (MHC) molecules, are well-documented, including the shrinking of the naïve T cell compartment due to thymic involution and the increase in the memory T cell pool [10]. In contrast, relatively little is known about the effects of aging on unconventional T cells, which are non-MHC restricted T cells that recognise non-peptide, non-polymorphic antigen-presenting molecules and express either αβ or γδ TCRs. The major subsets of unconventional T cells are γδ T cells, invariant Natural Killer T (iNKT) cells, and Mucosal-Associated Invariant T cells (MAIT) cells which collectively make up ∼10–30% of the T cell compartment in adults [11]. Unconventional T cells are dominant T cell populations especially at barrier sites such as the gut, skin and liver, and have been shown to be critical for the defense against a wide range of pathogens, particularly in early life before the expansion of conventional αβ T cells. They are also involved in cancer immunity, chronic inflammatory diseases, as well as tissue repair [12]. Thus, changes in unconventional T cells will have a wide effect on all aspects of aging and aging-related diseases.

Although unconventional T cells are often referred to as “innate-like” T cells, bridging the innate and adaptive immune systems due to their ability to acquire effector functions without prior exposure to antigens, it is becoming clear that not all unconventional T cells are “innate-like”. One of the hallmarks of “innate-like” unconventional T cells is their semi-invariant TCRs consisting of an invariantly rearranged TCR α or γ chain, combined with diversely rearranged β or δ chains, respectively [12]. iNKT cells, MAIT cells, and the majority of γδ T cells that express the Vδ2 chain is characterised by semi-invariant TCRs that allow them to recognise conserved microbial ligands and can be activated in a TCR-independent manner through cytokines, thus behaving in an NK cell-like, or “innate-like”, manner [13]. However, recently a subset of γδ T cells that express the Vδ1 chain has been shown to differentiate and undergo oligoclonal expansions like conventional, adaptive αβ T cells [14], [15]. Furthermore, “innate-like” conventional T cells called MHC II-restricted, innate-like, and commensal reactive T cells (T_MIC_) have also been described recently, with the ability to be activated by cytokines independently of their TCR [16]. Thus, T cells can broadly divided into unconventional or conventional T cells, within which they can be “innate-like” or “adaptive” [12].

In this Review, we will focus on the unconventional T cells from before birth to old age and summarise our current knowledge of what happens to them during aging, particularly highlighting how aging affects the "innate-like” and “adaptive” unconventional T cells differently. Then we will discuss how aging unconventional T cells may contribute to susceptibility to infections, reduced vaccine responses, and age-related pathologies.

## 2 γδ T cells

### 2.1 γδ T cell subsets and functions

γδ T cells are the first T cells to exit the thymus [17] and comprise a heterogenous group of cells that can be dated back to the evolution of jawed vertebrates, along with αβ T cells and B cells, and thus is the most ancient unconventional T cell subset [18]. Unlike αβ T cells, however, typical γδ T cells do not require MHC class I or class II molecules for recognizing antigens.

**Fig. 1:**
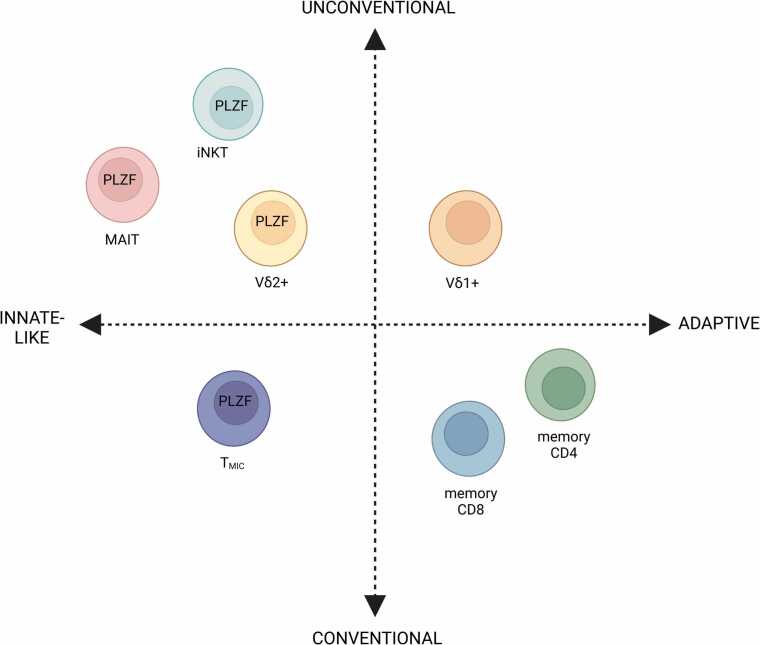
Conventional and unconventional T cells. T cells can be broadly grouped as conventional or unconventional based on their restriction molecules, while both groups of T cells can either be “innate-like” or “adaptive” depending on their innate-ness. Conventional T cells are MHC-restricted T cells that express TCRs composed of highly variable αβ chains, while unconventional T cells are non-MHC restricted T cells that recognise non-peptide, non-polymorphic antigen-presenting molecules and express either αβ or γδ TCRs. Within unconventional T cells, γδ T cells that express the Vδ2 chain are mostly Vγ9+Vδ2+ T cells which express the innate-like T cell transcription factor promyelocytic leukaemia zinc finger (PLZF) and are “innate-like” compared to γδ T cells that express the Vδ1 chain, which have been shown to have more adaptive biology. MAIT cells and iNKT cells also express PLZF, have a semi-invariant TCR and are considered “innate-like”. Within conventional T cells, memory CD4+ and CD8+ T cells are adaptive, while the recently discovered T_MIC_ (MHC II-restricted, innate-like, and commensal reactive T cells) [16] are “innate-like”, expressing PLZF and with the ability to be activated in a TCR-independent manner, despite being MHC class II-restricted. Created with Biorender.com.

**Fig. 2:**
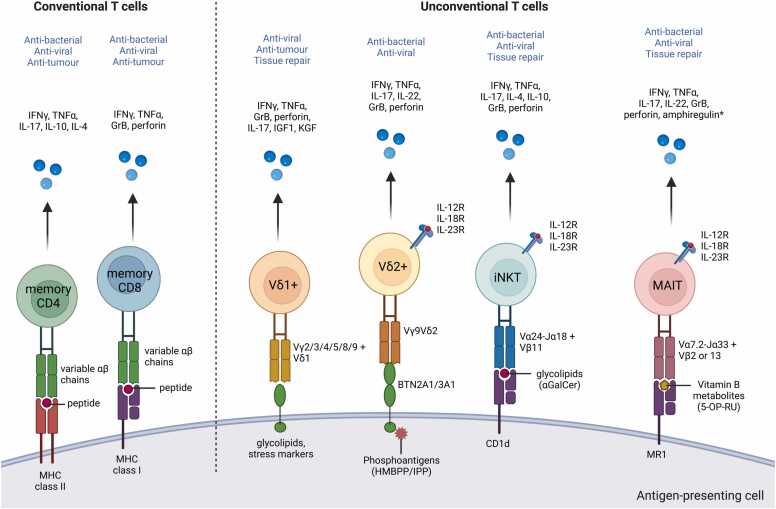
Ligands and functions of unconventional human T cells. Overview of ligands recognised by human γδ T cells, MAIT cells, and iNKT cells compared with conventional CD4+ and CD8+ memory T cells, and effector molecules and functions associated with each subset. CD4+ T cells express a TCR comprised of variable αβ chain combinations, which recognise peptides by major histocompatibility complex (MHC) class II molecules, while CD8+ T cells also express variable αβ chain combinations which recognise peptides presented by MHC class I molecules. Binding of the canonical phosphoantigen HMBPP to BTN3A1/2A1 induces conformational change that allows BTN2A1 to bind to the semi-invariant γδ TCR composed of Vγ9 chain and Vδ2 chain. iNKT cells recognise glycolipid ligands presented by CD1d molecules through their semi-invariant TCR composed of Vα14-Jα18 and Vβ11 chains. MHC-related molecule 1 (MR1) presents riboflavin (vitamin B2) metabolites and intermediates to MAIT cells, which express a TCR comprising an invariant Vα7.2-Jα33 chain and preferential use of Vβ2 or 13 chains. Vδ2+ T cells, iNKT cells and MAIT cells can also be activated in a TCR-independent manner solely through cytokine receptors such as IL-12R, IL-18R, and IL-23R due to their expression of promyelocytic leukaemia zinc finger (PLZF), producing IFNγ and/or IL-17, and can be considered “innate-like” T cells. The main effector molecules produced by each of these T cell subsets are listed, and the main protective functions of these cells are highlighted in blue. HMB-PP= (E)− 4-hydroxy-3-methyl-but-2-enyl pyrophosphate, BTN=butyrophilin. *Amphiregulin from MAIT cells has been demonstrated to promote wound healing in mice [203] and is upregulated at gene level in human MAIT cells upon TCR stimulation [200], but is yet to be demonstrated at the protein level in humans. Schematic based on [391]. Created with Biorender.com.

The main characteristic that defines γδ T cells is the expression of its distinctive TCR composed of a γ-chain and a δ-chain. The two main populations of human γδ T cells are the Vδ1+ and Vδ2+ T cells [19]. The Vδ1+ cells are found in the gut epithelium, skin, spleen and liver, while in the periphery, they make up to 30% of the γδ T cell population in healthy adults. They are mainly involved in maintaining epithelial tissue integrity, but they also have a role in anti-viral immunity, particularly in cytomegalovirus (CMV) control [20]. In addition, they have been shown to recognise B7-H6 expression on tumour cells via NKp30, suggesting they play a role in tumour control as well [21]. The Vδ1 chain is paired with various Vγ family members (Vγ2/3/4/5/8/9). Interestingly, the Vδ1+ T cell population has recently been described to be more adaptive compared to their “innate-like” Vδ2+ counterparts, based on the presence of highly clonal expansions in healthy control subjects and allogeneic-hematopoietic stem cell transplant patients after reactivation of CMV [14], [22]. This expansion is also observed in human immunodeficiency virus (HIV) and cancer, and show differentiation from a naïve to effector phenotype, demonstrating adaptive immunobiology [15]. Conversely, the Vδ2 chain is typically paired with the invariant Vγ9 chain, with some exceptions [23]. This semi-invariant TCR endows Vγ9+Vδ2+ T cells with “innate-like” biology where they acquire their rapid effector functions during fetal development and can be activated in a TCR-independent manner by cytokines, due to the expression of the master transcription factor of “innate-like” T cells, promyelocytic leukemia zinc finger (PLZF) [24], [25] (Fig. 1). They respond to microbial antigens such as hydroxy-methyl-butyl-pyrophosphate (HMBPP) or endogenous ligands such as isopentenyl pyrophosphate (IPP) through their TCR, which is an intermediate of the mevalonate pathway in mammals that can accumulate in transformed cells during tumorigenesis [26], [27] (Fig. 2). Vγ9+Vδ2+ T cells represent the largest γδ T cell subset in the human peripheral blood, encompassing 50–95% of γδ T cells [24], [25]. In addition, there are Vδ3+ T cells in the periphery which only account for 0.2% of γδ T cells, while in the liver they are more abundant [28], however relatively little is known about these cells as most studies have focused on Vδ1+ and Vδ2+ subsets.

In mice, γδ T cells are traditionally classified based on their γ chain expression, which are associated with their tissue of residence. Vγ1+ and Vγ4+ γδ T cells seem to have similar features with human peripheral blood γδ T cells, found mainly in lymphoid tissues and associated with the production of various cytokines such as IL-4 and IL-17 [29], [30]. In the epidermis, Vγ5+Vδ1+ T cells make up most dendritic epidermal T cells (DETC), where they can be activated by the butyrophilin-like molecule Skint-1 expressed by keratinocytes [31], [32]. The Vγ6+ subset produces IL-17 and IL-22 and migrate to the mucosal epithelia such as dermis, lung and female genital tract, while Vγ7 cells home to the intestine [30], [33]. The Vγ chain is also highly connected to their effector function, as γδ T cells expressing Vγ4 or Vγ6 cells preferentially produce IL-17, while Vγ1, Vγ5, or Vγ7 cells mainly produce interferon-gamma (IFNγ) [34]. These γ chains, paired with their respective δ chains, make up the murine γδ TCR which allows them to recognise diverse ligands such as Qa-1 [35] and MHC class Ib molecules T10 and T22 [36], [37].

The dominance of these cells differs hugely between species, with some animals such as cattle and pigs having much larger γδ populations within T lymphocytes [38], [39]. In humans, γδ T cells are a smaller population, constituting 1–5% of T cells in the adult peripheral blood and lymphoid tissues (including thymus, spleen, tonsil and lymph nodes), but they can expand dramatically (up to 60% of total T cells) in the blood of patients during infections [17]. They also make up 5–40% of intestinal intraepithelial lymphocytes (IEL) cells in humans [17], [40]. In mice, they are a more dominant T cell subset, comprising 1–4% of T cells within the thymus, secondary lymphoid organs and lung [17], and found in abundance in mucosal sites – 20–40% of intestinal IEL cells [41], 10–20% of all T cells in the female reproductive organs [42], and 50–70% of skin dermal T cells [43], [44], [45]. The majority of γδ T cells are CD4-CD8- (double negative; DN), although some CD8+ and a very rare CD4+ γδ T cell population have been reported [46].

### 2.2 γδ T cell development and early life

In humans, γδ T cells first develops in the fetal liver at 5–7 weeks gestation, then they are observed in the thymus at 8 weeks gestation [47], [48]. The Vγ9+Vδ2+ T cell subset is among the first T cell subset to be developed in the fetus, dominating the γδ repertoire during the second trimester and acquire the ability to produce IFNγ in response to HMBPP stimulation [49], [50]. However, the Vδ1+ population increases during gestation and the Vγ9‐Vδ1+ cells become the larger γδ T cell population at birth, populating the postnatal thymus, gut and skin [40], [51], [52]. Following birth, there is an immediate expansion of Vγ9+Vδ2+ T cells, which make up around 90% of the γδ T cell population within 4 weeks of age [53]. By one year of age, almost all Vγ9+Vδ2+ T cells have acquired a memory phenotype, can rapidly produce IFNγ, are cytotoxic and reach proportions similar to adults at 6 years of age [54]. Thus, Vγ9+Vδ2+ T cells are considered pre-programmed fetal-derived effector cells, which compensates for the delayed maturation of the αβ T cell compartment and provides early protection in neonates [49]. In contrast, the Vδ1+ T cell TCR is highly diverse in cord blood, but their TCR repertoire becomes more restricted into adulthood, responding to CMV infection via clonal expansions and behaving more “adaptive-like” [55]. Vδ1+ are predominantly associated with a role in tissue immunity, located in the skin, lung, intestine, and colon epithelia, compared to the Vδ2+ which dominate the blood [15], [23].

In mice, γδ T cells are the first T cells to exit the thymus. Vγ5+Vδ1+ DETCs are the first T cells to develop before birth and migrate to the skin epithelium and are key producers of IFNγ and growth factors [32]. This is followed by Vγ6+Vδ1+ T cells, which produce IL-17 and can be found in tissues such as the lung, liver and intestinal lamina propria [56]. More diverse Vγ1+ and Vγ4+ γδ T cells develop from late fetal life onwards in the fetal thymus and around the time of birth, including the Vγ4+Vδ1+ cells which migrate to the genital tract, the tongues and the lungs after birth [57], [58]. Fetal-derived γδ T cells are considered “innate-like” due to their reduced TCR sensitivity and rapid functional responses to innate stimuli like cytokines or pathogen-associated molecular patterns [59], [60]. Recent evidence suggests that the strength of TCR signaling in the thymus is the critical determinant of their effector function [33], as a strong TCR signal promotes an IFNγ-producing lineage (γδ1) while a weak TCR signal promotes an IL-17-producing lineage (γδ17) [61], [62], [63]. Uniquely, murine γδ17 cells can produce both IFNγ and IL-17 under certain circumstances [64], [65], and can differentiate from naïve γδ T cells into mature γδ17 cells in the periphery [66].

### 2.3 γδ T cells in aging

Following their expansion in childhood, there have been several reports of an age-dependent reduction in human γδ T cell frequencies and absolute numbers in the periphery [67], [68], [69], [70], starting to decrease between age 20–30 [30]. Total γδ T cells made about 2% of the total T cell population in blood in a recent large study of 203 healthy adults of different age groups and their frequency was higher in younger donors compared to the elderly, negatively correlating with age [71]. This has also been observed in a recent single cell analysis of immune cells in aging [72]. In terms of function, γδ T cells in the elderly have been reported to be more activated as seen from their expression of CD69, and produce TNFα at a higher level, but have an impaired proliferative response to stimuli such as IPP in the elderly and centenarians [67], [69], [73], [74]. They have also been shown to have an enhanced sensitivity to undergo apoptosis induced by anti-Fas [69], [75]. Interestingly, the age-dependent contraction of γδ T cells seems to be limited to the “innate-like” Vγ9+Vδ2+ population, while Vδ1+ T cells are maintained, or expanded, upon aging [76], [77], [78]. Furthermore, this loss seems to be sex-dependent, as there was a more profound loss of peripheral Vγ9+ T cells in men compared to women of the same age after their teenage years [79]. This loss of Vγ9+Vδ2+ cells in men was specific to effector memory (T_EM_) and effector memory cells re-expressing CD45RA (T_EMRA_), leaving central memory (T_CM_) cells as the predominant Vγ9+Vδ2+ cells in blood, while in women these proportions remained the same with aging. Functionally, as expected from their T_CM_ phenotype, the Vγ9+Vδ2+ T cells in older men had increased proliferative capacity and reduced IFNγ secretion upon IPP stimulation [79]. The biological reason for the reduction in Vγ9+Vδ2+ cells in the periphery with age, particularly in men, is still unclear. It has been shown that IL-15 stimulation induces proliferation in Vδ1+CD27‐/low cells [55], while IL-15 induces a high cell death rate on Vδ2+ T_EMRA_, which had low anti-apoptotic B-cell lymphoma 2 (Bcl-2) protein expression [80]. Thus, differences in susceptibility to activation-induced cell death (AICD) and responsiveness to homeostatic cytokines may account for the differential impact of aging on Vδ1+ and Vδ2+ subsets, although the difference in Bcl-2 expression is modest [77].

Differences in γδ T cell subsets between donors of different ages are more marked in CMV-infected individuals [78], [81]. As the Vδ1+ subset is the main subset involved in anti-CMV immunity [82], [83], there is a significant age-associated accumulation of terminally differentiated Vδ2- T cells and a lower Vδ2+/Vδ2‐ ratio, but this association was only found in CMV-seropositive donors. Indeed, CMV seems to accelerate the differentiation of γδ T cells from naïve to effector cells, and lead to a senescent phenotype, with increased CD57 and PD1 expression and lower IL-7Rα and Killer Cell Lectin Like Receptor G1 (KLRG1) expression [84], [85]. The accumulation of terminally differentiated and senescent Vδ1+ cells with age and CMV history is similar to the aging of CD8αβ T cells. This may be expected from the fact that Vδ1+ cells are more “adaptive-like” and their phenotype and function is influenced by stressors encountered during life, while Vδ2+ cells are more “innate-like” due to their semi-invariant TCR and exhibit a relatively stable distribution of differentiation phenotypes over time [70], [86], [87]. A recent twin study highlighted these differences, showing that Vδ1+ immune phenotype was influenced by the environment while Vδ2+ immune traits were more influenced by heritability [88]. Vδ2+ T cells also show hypo-methylated DNA and express genes associated with DNA damage repair, which may allow them to be more resistant to cellular senescence compared to Vδ2- T cells [89]. However, although CD57+Vδ2+ cells do not increase with age, these cells have been found in the blood and liver in elderly liver metastatic colorectal cancer patients undergoing chemotherapy, compared to younger patients [90], and suggests that external stressors such as chemotherapy and infections may accelerate the immunosenescence and functional impairment in Vδ2+ T cells with age.

In mice, there has also been reports of shifting Vγ/Vδ usage with age, similar to humans. Aging has been shown to lead to increased infection-induced mucosal adaptive Vγ4+ T cells in mice during *Listeria monocytogenes* infection [91], as well as during West Nile Virus (WNV) infection, which was associated with a higher mortality to WNV encephalitis in aged mice [92]. This increase in Vγ4+ T cells with age has a resemblance to human Vδ1+ cells, which similarly are expanded or maintained with age [93]. A recent study into the cellular composition of γδ T cells in peripheral lymph nodes showed that aging does not affect the function and global gene expression of γδ T cell and γδ TCR diversity [94]. However, aging was shown to alter δ chain usage: in IFNγ-secreting γδ1 T cells, there was an increased use of Vδ6 in Vγ1+ T cells with age, while Vδ2 was less favoured. In Vγ4+γδ1 T cells, usage of Vδ7 was slightly reduced. Significantly, IL-17-producing γδ17 T cells dominated the γδ T cell pool in aged mice [94]. This was due to the selective expansion of Vγ6+ T cells and augmented polarization of Vγ4+ T cells, mediated by increased IL-7 expression in the T cell zone of old mice. Expanded γδ17 cells were activated in the tumour-draining lymph node and migrated into the tumour microenvironment, and their infiltration correlated with tumour size in aged mice [94]. Thus, increases in IL-17-producing γδ T cells with age may lead to a bias towards the pro-tumorigenic γδ17 lineage during aging. This age-dependent accumulation of IL-17-producing γδ T cells has also been found in lungs [95] and adipose tissue [96], [97]. In some cases, IL-17 production was protective, however, as IL-17-producing Vγ4+ and Vγ6+ T cells increased in the lungs of aged mice and were necessary for the longer survival of aged mice after lung melanoma challenge [95]. The increase in γδ T cells with age in this study was limited to the lungs, while they significantly decreased in the spleen [95], which is consistent with a recent a single-cell profiling study of aged mice tissues that demonstrated that γδ T cells are increased in the aged liver and lungs, while there is a decrease in Sox4+ γδ T cells in the spleen [72], [98]. Another recent single cell analysis of immune cells in tissues of aged mice also showed that unconventional T cells, as identified as αβ- T cells, increased in the lungs and lymph nodes of aged mice [99]. Increased IFNγ-producing γδ1 T cells have also been shown to increase with age in other organs, as hepatic γδ T cells expressing NK1.1 had increased cytotoxicity and IFNγ production in very old age in mice [100]. In contrast, γδ IELs have been shown to disappear in the gut of aged mice [101], [102], [103], while γδ T cells are reduced in the uterus in female aged mice [104].

## 3 iNKT cells

Another well-characterised unconventional T cell population is the iNKT cell subset, which express an invariant TCR α chain rearrangement of Vα14-Jα18 in mice [105] and Vα24-Jα18 in humans [106]. These α chains are paired with a restricted repertoire of TCRβ chains: in mice, the Vα14-Jα18 chain is paired with Vβ7, Vβ8.2, or Vβ2, while in humans the Vα24-Jα18 chain is paired with Vβ11 [105], [107]. This unique TCR enables iNKT cells to recognise endogenous and exogenous lipid antigens presented by the MHC-I like molecule CD1d, the prototypical glycosphingolipid antigen being α-galactosylceramide (α-GalCer) [108] (Fig. 2).

### 3.1 iNKT cell subsets and functions

Upon activation through their TCR, iNKT cells rapidly secrete immunomodulatory cytokines, including IFNγ, TNFα, and IL-4. Thus, following treatment with α-GalCer, iNKT cells produce large amounts of cytokines, undergo clonal expansion, and subsequently activate NK cells, neutrophils [109], macrophages, dendritic cells [110], [111], B cells, and T cells. Activated iNKT cells can also directly induce cell death in tumour cells and infected cells. iNKT cells can also be activated in a TCR-independent manner by innate cytokines such as IL-12 and IL-18, which is key in their role in CMV immunity [112], while microbial activation of iNKT cells has been shown to be dominated by IL-12 signaling, rather than CD1d-mediated signaling [113]. iNKT cells can be further divided into CD4+ and CD4- subsets that differ in their cytokine profile and cytotoxic capacity. CD4+ iNKT cells can secrete large amounts of IL-4, IL-13, and IL-10, while the CD4- subset are the dominant IFNγ-producers and the cytotoxic subset [114], [115]. Thus, according to their coreceptor expression, iNKT cells are thought to play different roles in health and disease; for example, liver CD4- iNKT cells are much more effective at mediating tumour rejection compared to their CD4+ counterparts [116]. Human iNKT cells can be either CD4+, CD8+, or CD4-CD8- (DN), while in mice iNKT cells are mostly CD4+ or DN.

More recently iNKT cell subsets have been categorized according to their transcription factor expression and cytokine expression profiles: NKT1 (T-bet+), NKT2 (PLZF)^high^, and NKT17 (Retinoic acid related orphan receptor gamma-t; RORγt+) subsets, analogous to the Th1, Th2, and Th17 subsets of CD4+ T helper cells [117]. NKT1 cells predominantly produce IFNγ, while NKT2 cells produce IL-4 and IL-13 [118], and NKT17 cells produce IL-17 and IL-22 [119]. In addition, smaller subsets such as NKT10 cells which produce IL-10 and express E4-binding protein 4 (E4BP4) [120], NKT_reg_ which express Forkhead box P3 (Foxp3) [121], and NKT follicular helper cells (NKT_FH_) which express Bcl6 [122], have also been identified. In C57BL/6 mice, NKT1 cells are highly abundant and are especially enriched in the liver, spleen and thymus, while NKT2 cells are found in the mesenteric lymph node, and NKT17 cells are enriched in the lungs as well as inguinal, axillary, and cervical lymph node [123]. Thus, iNKT cells are poised to act in various tissues, and the tissue-specific distribution of iNKT cell subsets affects their cytokine response which in turn modulates the outcome of immunity (reviewed in [124]). Less is known about the functional subsets of iNKT cells in humans, but DN and CD8+ iNKT cells in humans have been found to be similar to murine NKT1 cells, which are predominantly IFNγ-producers and have strong cytotoxic functions when activated [124].

Invariant NKT cells are found in various tissues around the body, but unlike conventional T cells, the majority of iNKT cells do not recirculate and establish long-term residency within tissues. In human peripheral blood, they are relatively infrequent, with a range from 0.001% up to > 1% of T cells [125]. Frequencies of iNKT cells in the human liver is similar to the blood [126], which is in stark contrast to mice in which iNKT cells can make up to 30–50% of all intrahepatic lymphocytes [127], [128]. In visceral adipose tissue (VAT), iNKT cells comprise 15–20% of total T cells in both humans [129] and mice [130], while in the murine lung they are around 5% of resident lymphocytes [124].

### 3.2 iNKT cells development and early life

The innate-like properties of iNKT cells are imprinted during their development. iNKT cell develop in the thymus from precursor CD4+CD8+ (DP) thymocytes and are positively selected by DP cortical thymocytes expressing CD1d, rather than cortical epithelial cells that select conventional MHC-restricted T cells [131]. CD1d is highly expressed in the thymus and may present endogenous ligands such as α-GalCer, α-GluCer, and an α-linked monohexosylceramide, which have been found at trace levels on murine thymocytes [132], [133]. The strength of TCR signaling in the thymus commits the iNKT cells to either NKT1, NKT2, or NKT17 subsets [134], where a strong TCR signal strength promotes NKT2 and NKT17 development and upregulation of the iNKT cell master transcription factor, PLZF. PLZF expression in iNKT cells immediately after positive selection allows iNKT cells to acquire their “innate-like” effector phenotype and tissue-homing properties before exiting the thymus and regulates the gene expression of transcription factors T-bet, GATA binding protein 3 (GATA3), and RORγt [135], [136].

Following development in the thymus, murine iNKT cells exit as an immature, but not naïve, subset [137], where exposure to the microbiota early in life controls their development and tissue accumulation [138]. iNKT cells in germ-free mice have been shown to be less differentiated and respond poorly upon antigen stimulation, suggesting that commensal bacteria can drive the maturation of iNKT cells [139]. Furthermore, the presence of commensal bacteria following birth restricts the accumulation of iNKT cells in the colon and lungs and was found to reduce the susceptibility to experimentally induced colitis and allergic asthma [140]. Microbial exposure 2 weeks after birth was insufficient to minimize the accumulation of iNKT cells in the colon and experimentally induced colitis in adult animals, suggesting that the microbiota can modulate the development of iNKT cells within the first 2 weeks of life [141]. Thus, microbial colonization during the neonatal period imprints the abundance of iNKT cells in barrier tissues, which can have long-term effects on susceptibility to infection and inflammatory diseases [138].

In humans, iNKT cells are present in the human fetal thymus at higher frequencies at the beginning of the second trimester (between 12 and 20 weeks of gestation) [142], but they gradually decline with gestational age, and are present at a very low frequency in the post-natal thymus, suggesting that iNKT cells exit the thymus largely during early fetal development. iNKT cells were found in the small intestine by 18 weeks of gestation and increased with gestational age, making up to 5.4% of the total T cells [125]. In comparison, in the fetal lung, spleen, and mesenteric lymph nodes they made up less than 0.5% of the total T cells in each tissue [125]. iNKT cells in the fetal thymus were largely immature with a CD4+CD161- phenotype, while iNKT cells in the fetal small intestine had a more differentiated phenotype, with higher frequencies of CD4-CD161+CD45RO+ cells and the ability to produce IFNγ upon activation, suggesting that they differentiate *in utero* in the absence of commensal microflora. In cord blood, iNKT cells remain mostly CD4+and 50% express CD161, as well as naïve markers C-C chemokine receptor 7 (CCR7) and CD62L [143]. Following birth, the absolute number and frequency of iNKT cells seem to be relatively stable, with no significant difference observed between cord blood, children, and young adults [144].

### 3.3 iNKT cells in aging

There have been various reports of a reduction of iNKT cell frequency and absolute numbers with advancing age in humans [126], [145], [146], [147]. One of the first studies in humans showed CD3+ cells from healthy elderly individuals had a decreased percentage of Vα24+ T cells compared with younger donors [148]. Although this study would have included Vα24+Vβ11‐ non-iNKT cells, subsequently a significant correlation of Vα24+Vβ11+ iNKT cell frequency with age in healthy donors was confirmed [147], [149]. Similarly, an age-dependent decrease in circulating Vα24+Vβ11+ iNKT cell numbers was confirmed in both healthy controls and cancer patients, and this decline was found to be faster in males compared to females [150]. Gender seems to have a significant effect on iNKT cell frequencies [151], and a male-specific decline in CD1d‐tetramer+iNKT cell frequencies with age was also reported in a Korean cohort by Kee et al., where there was no significant effect of age on iNKT cell frequencies in the total cohort or in females but was significant in males [152]. In contrast a recent large Caucasian study found the opposite, where the iNKT cell frequency overall or in males did not correlate with age but significantly declined with age in the females [71], [153]. Finally, there are also some studies that did not observe a significant effect of age on Vα24+Vβ11+ iNKT cells [154], [155], [156]. Given that iNKT cells are present at a very low frequency in humans, around 0.01% of all T cells in the blood [71], there is likely a large variability between studies depending on the sample size and method of iNKT cell quantification (CD1d tetramer+, Vα24+Vβ11+, or CD3+6B11+, which recognises the Vα24-Jα18 CDR3 loop) [144]. Early studies identifying iNKT cells as CD3+CD56+ cells [157] or CD3+Vα24+ cells in relation to age will likely include many conventional memory CD8+ T cells and MAIT cells. Thus, there is some indication from recent gender-controlled studies that iNKT cell frequencies may decline in the elderly in a gender-dependent manner, but further research is necessary to confirm this.

In terms of changes in functionality with age, two early studies have found that the rapid reduction in iNKT cells with age was associated with an increase in the CD4+ subset and a decrease in the DN subset [148], [152]. Cytokine profile seems to be affected by age as well, with reduced IFNγ production and a shift from a Th1 to Th2 cytokine profile with aging [145], [150]. There is conflicting evidence on the effect of aging on the proliferative capacity of iNKT cells, as in one study iNKT cells from elderly donors showed impaired proliferation in response to α-GalCer and IL-2 stimulation [147], while in a different study it was demonstrated that α-GalCer stimulation resulted in the rapid expansion of iNKT cells from healthy elderly donors and there was no effect of age on fold expansion in response to α-GalCer [149]. Thus, there seems to be a reduction in the DN, IFNγ-producing iNKT cell subset with age, but the significance of these changes in larger cohorts and in age-associated diseases need to be confirmed.

Early studies in mice looking at “NKT” cells in aging identified these cells as NK1.1+TCRαβ cells and found these cells to increase in the liver until middle age and decrease thereafter [158], [159]. However, we now know this NK1.1+TCRαβ cell population would have included not only CD1d-restricted iNKT cells but a mixed population of NK-cell receptor expressing CD8+ T cells and MAIT cells, and thus cannot be classified exclusively as iNKT cells. Examining classical iNKT cells, the frequency of CD1d-tetramer positive iNKT cells in the spleen of aged mice was three-fold higher compared to younger mice [160]. In terms of function, they seem to have increased function which contributes to age-associated decline in the adaptive T cell immunity, as age-associated increase in IL-10 from splenocytes, impaired T cell proliferation, and antigen-specific delayed-type hypersensitivity were prevented by anti-CD1d monoclonal antibody treatment in vivo [160]. Augmented responses of liver iNKT cells to α-GalCer and CpG stimulation in older mice have also been reported, leading to an age-dependent increase of TNFα levels and FasL expression on CD1d dimer+ iNKT cells in these mice, which led to multi-organ dysfunction syndrome (MODS) and a high mortality in the aged mice [161], [162]. In addition to changes in responsiveness, changes in iNKT cell subsets with age has been reported, particularly in the thymus, where iNKT cells showed reduced proliferative capacity [163], [164], [165], [166]. [166]Notably, hepatic iNKT cells from aged mice produced higher levels of IL-17 compared to young iNKT cells, and adoptive transfer of aged iNKT cells into young mice resulted in hepatic injury [166], [167]. Furthermore, Herpes Simplex Virus-2 (HSV-2) infection in mice led to significantly higher levels of IL-17 in older mice, compared to younger mice, and was associated with increased neutrophil recruitment to the liver and chemokine production, and mortality [166]. It is important to note, however, that different strains of mice may have different age-dependent effects on iNKT cells, as it has been reported that B6 mice are skewed towards IFNγ-producing NKT1 cells at all ages of mice in the thymus and have a relatively stable frequency of iNKT cells, compared to BALB/c mice, where the frequency of thymic iNKT cells increased with age (up to 20 weeks) and were skewed toward IL-4-producing NKT2 cells up to 8–10 weeks of age, after which NKT1 and NKT17 cells predominated [165].

Interestingly, although iNKT cells are mostly considered to be tissue-resident, a new, circulating CD244+CXCR6+ iNKT cell subset was recently identified, which showed NK-like features such as high IFNγ and high cytotoxicity compared to CD244‐CXCR6+ iNKT cells, which were more tissue-resident as shown in parabiosis experiments [168]. These CD244+CXCR6+ iNKT cells were found to be enriched in mucosal tissues such as the lung and lamina propria of the intestine. Significantly, these cells were found at a higher frequency in young mice but lower in middle-aged mice (up to 48 weeks), and they protected mice from tumour metastasis of melanoma cells and promoted anti-viral immune responses against influenza virus infection [168]. The proportion of human iNKT cells expressing CD244+CXCR6+, which were human counterparts to the murine cells by gene expression analysis, was significantly lower in older donors. It would be important to confirm this finding in a larger study of various age groups and to see whether their anti-tumour and anti-viral functions are retained in the elderly.

## 4 MAIT cells

MAIT cells are defined by their expression of a semi-invariant TCR consisting of the canonical TCR α chain, Vα7.2-Jα33 in humans, and Vα19-Jα33 in mice, which is preferentially paired with Vβ2, Vβ13.2, and Vβ22 in humans, or Vβ6 or Vβ8 in mice [169], [170], [171]. This TCR allows MAIT cells to recognise the highly evolutionarily conserved, MHC class I-related protein 1 (MR1) [172]. The nature of the ligand presented by MR1 was discovered by Kjier-Nielsen et al. [173], who found that metabolic byproducts of the riboflavin (vitamin B2) biosynthesis pathway could potently activate MAIT cells. Thus, MAIT cells can be activated by organisms possessing the riboflavin synthesis pathway, including *Mycobacteria*, *Enterobacter*, *Pseudomonas*, *Salmonella*, and *Candida* species, but not those lacking this pathway (e.g., *Streptococcus pyrogenes* and *Enterococcus faecalis*) [170], [173], [174]. An early intermediate of the riboflavin pathway, 5-(2-oxopropylideneamino)- 6-D-ribitylaminouracil (5-OP-RU) was subsequently shown to be the true activating MR1 ligand [175], [176], [177] (Fig. 2).

### 4.1 MAIT cell subsets and functions

In humans, MAIT cells are the most dominant unconventional T cell subset in the blood and tissues, with MAIT cells making up 1–10% of total T cells in the blood, up to 60% of CD4- T cells in the jejunal mucosa [178], [179], 10% of T cells in the colon [180], 20–50% of T cells in the liver [169], [181], [182], 2–4% of T cells in the placental intervillous blood [183], and 2–4% of T cells in the airway and lungs [184]. In comparison, they are relatively low in frequency in the female genital tract [185] and lymph nodes [169]. In contrast to the abundance of MAIT cells in human tissues, MAIT cells are rare in commonly used laboratory strains of mice, and thus many murine studies have used Vα19i-transgenic mice [186]. Recently, characterization of wild-type murine MAIT cells with MR1 tetramers showed an enrichment of MAIT cells in the lung (mean 3.3% in T cells in C57BL/6 mice), liver (0.6% of T cells) and lamina propria (0.7% of T cells), but they were rare in peripheral blood (<0.1% of T cells) [187], [188]. MAIT cells can be separated into subsets based on CD4 and CD8 expression, and in human blood, the majority of MAIT cells are CD8+ T cells, while DN MAIT cells constitute about 15%. In C57BL/6 mice, the DN MAIT cells are the main population [188]. Human MAIT cells are also characterized by high expression of the C-type lectin-like receptor, CD161, and CD161++Vα7.2+ T cells have been shown to overlap with the cells stained by the MR1-tetramer [178].

Following stimulation through their TCR, MAIT cells can rapidly secrete cytokines and have potent cytotoxic potential [189]. Human peripheral blood MAIT cells predominantly secrete IFNγ and TNFα upon activation, but can also produce IL-17 due to their constitutive expression of RORγt. In certain tissues, MAIT cells are skewed towards IL-17 and IL-22 production, such as in the liver [181], where they are the dominant IL-17-producing T cell population, and the female genital tract [185]. In addition to TCR-mediated activation, MAIT cells can be activated in a TCR-independent manner due to their high cytokine receptor expression, rapidly releasing IFNγ in response to proinflammatory cytokines such as IL-12 and IL-18, Type I IFNs, IL-15 and tumor necrosis factor-like cytokine 1A (TL1A) [190], [191], [192], [193]. This innate-like ability allows them to respond to toll-like receptor (TLR) agonists, or bacteria which lack the riboflavin synthesis pathway such as *E. faecalis* [190], [194]. Significantly, MAIT cells be activated by viruses in a TCR-independent manner [191], and contribute to protection against lethal influenza virus infection in mice [195] and humans [196]. MAIT cells can also directly kill infected cells [197] as well as tumour cells [198], which can be mediated in an TCR-dependent or TCR-independent manner [199].

Recently, a new function of MAIT cells in tissue repair was discovered simultaneously by several groups [193], [200], [201], [202]. Transcriptomics studies showed that MAIT cells expressed a tissue repair signature that was similar to that of murine H2-M3 restricted commensal-specific Tc17 when activated by their TCR, but not by TCR-independent activation [193], [200], [201]. Strikingly, human MAIT cells activated by *E. coli* supernatants accelerated wound closure in an in vitro wound-healing assay [193], and directly applying 5-OP-RU in vivo on wounded skin was sufficient to accelerate tissue repair in mice [202], mainly by secreting amphiregulin and promoting keratinocyte proliferation [203]. These results suggest that MAIT cells have a previously unrecognized role in barrier integrity maintenance and repair (reviewed by [204]) (Fig. 2).

### 4.2 MAIT cell development and in early life

MAIT cells have been shown to be selected in the thymus [171], [205], by MR1 expressed on DP thymocytes [206], [207]. Like iNKT cells, MAIT cells require PLZF for their development, as PLZF-deficient mice lacked MAIT cells [11], [188]. Intrathymic development of murine MAIT cells can be divided into three developmental stages based on the expression of CD24 and CD44 in mice, or CD27 and CD161 in humans [208], [209]. A recent analysis of murine unconventional T cells by single-cell RNA-seq analysis demonstrated the development of MAIT1, MAIT2, and MAIT17 subsets in the thymus, which was analogous to NKT1, NKT2, and NKT17 development [210], although MAIT2 cells were mainly developmental intermediates of MAIT1 and MAIT17. However, significantly MAIT cells in humans do not separate into MAIT1 or MAIT17 lineages and can co-express RORγt and T-bet in mature MAIT cells [210], suggesting there is more plasticity in human MAIT cells than mice. Unlike iNKT cells, murine and human MAIT cells exit the thymus as naïve cells [205]. In contrast to the intrathymic expansion of iNKT cells, which occurs unaffected in germ-free mice, expansion of MAIT cells is dependent on microbial colonization and occurs in the periphery, as MAIT cells are undetectable in germ-free mice [172]. Reconstitution of germ-free mice with a single strain of bacteria that possesses the riboflavin synthesis pathway leads to the expansion of MAIT cells [174].

Human MAIT cells are programmed early during development to reside in mucosal sites, with low expression of lymphoid homing markers such as CD62L on MAIT cells in the thymus and cord blood [206]. Second trimester fetal MAIT cells are already functionally mature and enriched in the small intestine, liver and lung, expressing higher levels of PLZF and CD45RO compared to those in the lymphoid tissues [179]. This was associated with the ability to proliferate and secrete cytokines in response to *E. coli* infection [211]. Developing MAIT cells thus increase their PLZF expression post-thymically, but prior to microbial colonization, which is required for the acquisition of their memory phenotype and innate reactivity to bacteria [211]. The fetal MAIT cell coreceptor expression also changes during gestation, with transition from expression of CD8αβ to CD8αα [209], [211], as well as the expansion of the DN subset coinciding with the contraction of the CD8+ subset, suggesting that DN MAIT cells may be derived from CD8+ MAIT cells [212].

Human MAIT cells are naïve cells in cord blood, with an immature phenotype, low CD161 expression and a higher proportion of CD4 coreceptor expression [11]. MR1-tetramer+ cells make up only 2–15% of Vα7.2+CD161++ T cells in cord blood, but this proportion increases very rapidly after birth, with already more than 50% of Vα7.2+CD161++ T cells staining for the MR1-tetramer at 1 month of life, and this rising to 100% by 1 year of life [213]. This suggests that MAIT cells expand rapidly after birth, and acquire CD161 expression during early childhood, probably due to exposure to microbial-derived MR1 ligands. 20% of MAIT cells express CD69 by 3–4 weeks of age, suggesting a recent activation in vivo, and acquire a memory phenotype soon after birth as most of them are CD45RO+ cells by 3 months of age, losing CD8β expression concomitantly [178], [179], [205]. The frequency of MAIT cells reach adult proportions between 2 and 6 years of age [209], [213], [214], although the exact dynamics may differ depending on geographic location [215]. These mainly central (CD27+CD45RA-) and effector memory-like (CD27-CD45RA-) profiles are sustained into adult and old age [11].

### 4.3 MAIT cells in aging

There have been several studies showing that human MAIT cell frequencies and absolute numbers in the periphery decrease in the elderly and is negatively correlated with age [11], [214], [216], [217]. Early reports in Scandinavian and British cohorts showed that MAIT cell frequency increases with age, reaching maximum numbers in their twenties [217], [218]. Then there is a progressive decline in MAIT cell frequency with age, with individuals that are > 80 years having about 10 times less MAIT cells, both as absolute numbers as well as frequency within T cells, than donors of fertile age [218]. These results, which identified MAIT cells as CD161++Vα7.2+ T cells, were confirmed definitively using MR1-tetramers, showing MAIT cells increased from birth to about 25 years of age, and declined thereafter [219]. Age-dependent reduction in MAIT cell frequency was also confirmed in studies of healthy controls [11], [214], [216] as well as in patients with cirrhotic liver disease [220] and gastric cancer [221]. The reduction of MAIT cells with age may depend on the gender, as the loss of MAIT cells in the elderly has been reported to be slower in females compared with males [214], [216], [222], with a significantly higher frequency of MAIT cells in women of reproductive age compared to men of the same age [218]. The frequency of MAIT cells expressing the apoptosis marker Annexin V correlated with age, as well as MAIT cells expressing the activation marker CD69 [214], suggesting increased activation and apoptosis may be contributing to the reduction in MAIT cells with age.

Several studies have demonstrated that there is a specific decrease in the CD8+ MAIT cell population in old age, compared to the DN MAIT cell population. Novak et al. showed that there is a gradual decrease in the CD8+ /DN MAIT cell ratio with age, and while the percentage of CD8+ MAIT cells is relatively homogenous among children (about 80%), there is a higher interindividual variability in the percentage of CD8+ subset within MAIT cells with increasing age (ranging between 5% and 80% in the centenaries) [218]. This is consistent with other studies showing similar age-dependent reduction in CD8+ MAIT cells while the DN MAIT cell population remained constant [212], [216], or declined slower [219]. As CD8+ MAIT cells are functionally superior to DN MAIT cells in both MR1-dependent and MR1-independent stimulations in vitro, with higher cytokine expression and cytolytic molecule expression, and DN MAIT cells are more prone to apoptosis [212], [223], this change in CD8+ /DN MAIT cell ratio may influence the overall functionality of MAIT cells with age. Lee et al. also found an increase in CD4+ MAIT cells in the elderly compared to young donors [216]. In contrast, however, Loh et al. did not observe a change in CD4, CD8, DN, and DP subsets with increasing age, and showed that despite the decline in MAIT cell numbers, MAIT cell clonotypic expansions remain prevalent in aged individuals [11].

Despite changes in absolute numbers and subset frequencies with age, there is evidence that MAIT cells retain their effector functions in old age. A recent study has shown that MAIT cells in the elderly had significantly higher expression of Granzyme B (GrB), IFNγ, and CD107α at baseline, and suggested that there is low-grade basal inflammatory activation of MAIT cells in old age [11]. Due to the high basal expression of these molecules, fold change increase in GrB or IFNγ upon in vitro stimulation with *E. coli* or influenza-infected lung epithelial cells was reduced but removing the MAIT cells from the inflammatory aged milieu restored normal levels and pathogen-specific activation [11]. Furthermore, MAIT cells from elderly donors were also found to have significantly higher levels of IFNγ, IL-17, and GrB in response to phorbol myristate acetate (PMA)/ionomycin compared to young adults [214]. Interestingly, one study found that CD8+ MAIT cells produced significantly more IL-4 in older individuals compared to younger donors, but similar levels of IFNγ and IL-17 [216], suggesting there may be a slight shift in cytokine production with old age. Thus, MAIT cells retain their potent effector functions in old age but their overactivation due to inflammaging may impede their effectiveness.

It has yet to be seen If MAIT cells are recruited to the tissues with old age. One study found that MAIT cell frequencies in the lamina propria of the stomach were not significantly different between children, adults and elderly groups of donors [224]. Similarly, there was no significant correlation of pulmonary MAIT cell frequency with age in a small cohort [225], suggesting that MAIT cells loss in the blood is not associated with their accumulation in tissues.

Due to the low numbers of MAIT cells in laboratory strains of mice, there is very little information on how MAIT cell frequency and function change in aged mice. In a recent primate model of rhesus macaques, aging was associated with the loss of MAIT cells from the blood [226], like human MAIT cells. Further studies on wild-derived CAST/EiJ mice, where MAIT cells are 20 times more frequent than in C57BL/6 mice [227], may allow insight into changes in MAIT cells with age as well as their role in age-related diseases.

In summary, aging leads to the reduction in CD8+ MAIT cells in humans, which retain their effector functions but show low-grade basal activation due to the inflammatory aging environment.

## 5 Role of unconventional T cells in age-related diseases

Deficiencies and defects in unconventional T cells have been associated with susceptibility to bacterial and viral infections, as well as cancer, autoimmunity, and chronic inflammation, all of which are observed with increasing frequency in the elderly. The role of each unconventional T cell subset in these pathologies, without the context of age, has been extensively reviewed previously (MAIT cells [228], [229], iNKT cells [124], [230], and γδ T cells [231]), and a detailed discussion of each disease is beyond the scope of this Review. Therefore, in this section we will discuss the potential role of unconventional T cells in diseases associated with advancing age, focusing on their role in bacterial and viral infections, vaccine responses, cancer, and tissue homeostasis/repair, then discussing how age may affect these roles.

### 5.1 Role in bacterial infections and effect of aging

*Streptococcus pneumoniae* is the leading cause of pneumonia and meningitis in both children and the elderly. In mice, iNKT cells are essential for protection of mice from mortality from *S. pneumoniae* infection [232], [233], particularly the granulocyte-macrophage colony-stimulating factor (GM-CSF) produced by NKT17 cells [234], and γδ T cells are also involved in the early recruitment of neutrophils [235], [236]. Murine MAIT cells have not yet been shown to be essential for *S. pneumoniae* protection [234], but as the abundance of iNKT cells and MAIT cells are reversed in mice and humans, MAIT cells may play a much larger role in *S. pneumoniae* protection in humans than in mice. Indeed, in an experimental human challenge with *S. pneumoniae*, increased MAIT cell numbers at baseline correlated with protection against mucosal colonization [237]. A higher MAIT cell and Vδ2+ T cell infiltration into the airways was also associated with less severe community-acquired bacterial pneumonia [238].

Elderly individuals are vulnerable to develop tuberculosis, with a progressive increase in mortality with age in developed countries [239]. γδ T cells, particularly the Vγ9+Vδ2+ T cell population, are important in *Mycobacteria tuberculosis* (*M. tb) infections*, which expand following recognition of HMBPP, directly kill intracellular *M. tb* [240] as well as enhancing conventional T cell responses [241], [242]. Mice lacking MAIT cells have increased mycobacterial loads after mycobacteria infection [174], [243]. Mice lacking iNKT cells do not have increased susceptibility to *M. tb* infection [244] but administration of α-GalCer or adoptive transfer of iNKT cells contribute to the control of *M. tb* replication in vivo [245], [246]. MAIT cells, iNKT cells and Vγ9+Vδ2+ T cells as well as NK cells are specifically depleted in a T-bet deficient patient with mycobacterial disease [247], suggesting the lack of these unconventional T cells in the elderly may contribute to increased susceptibility to mycobacteria infections.

Aging is also associated with susceptibility to enteropathogenic *L. monocytogenes,* with a high mortality rate of 20% of infected individuals > 65 years old [91]. γδ T cells are protective in *L. monocytogenes* infections, where there is an age-dependent accumulation of Vγ4+ T cells [91]. iNKT cells have been suggested to be important for clearance of *Pseudomonas aeruginosa* from the lungs [248], as well as prevention of joint inflammation after *Borrelia burgdorferi* infection [249]. Mice lacking γδ T cells have impaired clearance of *P. aeruginosa* in the lung [250] and *Staphylococcus aureus* in the kidney [251], and Vγ9+Vδ2+ T cells were shown to mediate protection against *S. aureus* and *E. coli* in a chimeric severe combined immunodeficiency (SCID) mouse model [252]. Mice lacking MAIT cells succumb to *Klebsiella pneumoniae* infection [253], have delayed clearance of *Francisella tularensis* [254], [255], *E. coli* [174], and *Legionella longbeachae* infection [256]. A near complete deficiency of MAIT cells was found in a cystic fibrosis patient with severe lung bacterial infection, despite the lack of other overt immunodeficiencies and extensive use of effective antibiotics [257], suggesting that lack of MAIT cell is associated with severe susceptibility to bacterial infections.

Therefore, although the direct effect of aging in these infections has not been investigated, the loss of MAIT cells, Vδ2+ T cells, and possibly iNKT cells with age will most likely have a significant impact on the anti-bacterial host defense in the elderly and requires further investigation.

### 5.2 Role in influenza and effect of aging

Age is a major risk factor for mortality resulting from influenza. γδ T cells can inhibit virus replication by killing influenza-infected macrophages and lung alveolar epithelial cells [258], [259]. Mice lacking iNKT cells are more susceptible to influenza A virus (IAV) infections than control mice [260], [261], [262]. MR1-/- mice lacking MAIT cells had higher weight loss and mortality when inoculated with the highly pathogenic PR8 IAV strain, which was ameliorated by MAIT cell adoptive transfer [195], suggesting the loss of these unconventional T cells in old age may contribute to the susceptibility of these individuals to lethal flu. Indeed, elderly patients who succumbed to severe avian H7N9 influenza disease had blood MAIT cell numbers that were significantly reduced compared to age-matched controls [196], although it is possible that the MAIT cells had migrated to the lungs in these patients. In addition to the ability of these cells to directly kill infected cells, these unconventional T cells seem to facilitate and enhance the mounting of an effective antigen-specific adaptive immune response, promoting CD4+ T cell follicular helper cell differentiation and influenza virus-specific antibody production [263], as well as activating lung-resident NK cells [261]. In addition, tissue barrier maintenance and repair by unconventional T cells may be important in protection against influenza; for example, iNKT cells stimulated by IL-1β and IL-23 produced IL-22, which protected the lung epithelium from influenza-mediated damage [264]. In addition to the loss of these unconventional T cells in the blood with age, the ability of these cells to respond to influenza virus may wane in the elderly, as the upregulation of activation markers such as Human Leukocyte Antigen DR isotype (HLA-DR), CD69, and CD38 by pulmonary MAIT cells and γδ T cells in response to infection with influenza virus in vitro trended downwards with age, although this was not significant due to high variability between donors [225], and would be worth investigating further. Thus, as protective roles for unconventional T cells in flu has been demonstrated extensively, age-related changes in these cells are expected to have a huge effect on the immune response to flu in the elderly. Furthermore, these patients are also at greater risk of secondary bacterial infections and opportunistic infections, so to further elucidate the role of these cells in aging is of great clinical importance.

### 5.3 Role in COVID-19 and effect of aging

The COVID-19 pandemic caused by the severe acute respiratory syndrome coronavirus 2 (SARS-CoV-2) highlighted how the elderly population were most at risk for this serious infection. Unconventional T cells are generally reduced in the blood of severe COVID-19 patients [265], [266], [267]. MAIT cells are one of the T cell populations most significantly affected by COVID-19 and are profoundly depleted from the blood in comparison with age- and Body Mass Index (BMI)-matched controls [268], [269] and are recruited to the inflamed airways [270]. Significantly, their activation level as measured by CD69 levels was associated with poor clinical outcome and death in several studies [269], [270], [271]. In fatal cases of COVID-19, serum IL-18 levels correlated with MAIT cell activation [269]. γδ T cells are also reduced in the blood in patients hospitalized for COVID-19, compared to healthy controls, and were activated in the blood as indicated by CD69 and recruited to airway tissues [266], [267], [272]. Interestingly, the reduction in frequencies is specifically in Vδ2+ T cells but not Vδ1+ T cells [273], [274], and their frequency correlates with disease severity [274]. While no differences in Vδ1+ T Vδ1+ T cells have been identified, Vδ1+ T cells expressing CD160 was associated with moderate disease by single-cell analysis, suggesting they are protective in COVID-19 [268]. Furthermore, TCRδ1+ CDR3 sequences showed evidence of clonal focusing in COVID-19 patients aged > 50 years [275]. Finally, iNKT cells are also diminished in severe COVID-19 [266], [276]. How aging affects the response of unconventional T cells to COVID-19 remains to be investigated. Given the importance of unconventional T cells particularly in early life, and the low severity of COVID-19 in children, this avenue should be further explored [277]. The low-grade basal activation and cytotoxic phenotype of MAIT cells in the elderly [11] may contribute to further overt activation of MAIT cells and bystander cytotoxicity in the older population during COVID-19 [269].

### 5.4 Role in vaccine-induced responses and effect of aging

Unconventional T cells have been shown to play critical roles in the immune response to flu vaccination (recently reviewed for iNKT cells [278], γδ T cells [279], and MAIT cells [280]), but aging may affect the effectiveness of these cells in the vaccine response of the elderly. Indeed, although γδ T cells in aged individuals receiving the inactivated flu vaccine proliferated and produced IFNγ in response to restimulation [281], a recent study showed that a lower number of activated and proliferating γδ T cells were observed at baseline and following flu vaccination in the elderly, compared to young individuals [282]. Furthermore, proliferation levels of γδ T cells correlated with vaccination titer in the young, but not in the elderly, suggesting aging negatively impacts the role of γδ T cells in flu vaccine efficacy [282]. Importantly, this study showed that there was no significant difference in activation levels measured by CD38 between the young and old donors, and the frequency of proliferating γδ T cells as measured by Ki67 was higher in the elderly [282]. However, due to the reduced absolute cell count of γδ T cells in the elderly, the total number of activated and proliferating γδ T cells was lower in the elderly following flu vaccination [282], demonstrating how the reduction of γδ T cells in old age may lead to reduced vaccine responses.

There is evidence supporting a role for unconventional T cells in response to COVID-19 vaccination. MAIT cells have been shown to play an important role in the initial priming of CD8+ T cell immune responses to antigens encoded by the ChAdOx1 viral vaccine vector, as used in the COVID-19 vaccine, in both mice and humans [283]. Furthermore, MAIT cells may be associated with the adaptive immune response magnitude to the Pfizer-BioNTech mRNA vaccine against SARS-Cov-2, as recipients who had reduced MAIT cell frequencies and responsiveness showed lower immunogenicity [284]. The effects of aging on unconventional T cells in the context of vaccine responses to COVID-19 are still unknown, and it will be important to know whether the ability of MAIT cells to contribute to the priming of the adaptive immune system is retained in older recipients.

### 5.5 Role in cancer and effect of aging

The incidence of most cancer types increases with age. The anti-tumour potential of unconventional T cells, notably of iNKT cells, is well known, and has been extensively reviewed [230], [285], [286], [287], [288]. Briefly, reduced iNKT cell frequencies in humans are associated with poor prognosis in cancer patients, including head neck carcinoma [289], acute myeloid leukemia [290], neuroblastoma [291] and chronic lymphocytic leukemia [292]. A higher degree of iNKT cell infiltration in colorectal cancer patients was associated with improved survival [293], and therapeutic activation of iNKT cells via α-GalCer increases anti-tumour immunity and inhibits tumour progression [294]. Additionally, MAIT cells have also been shown to enhance anti-tumour immunity in the presence of 5-OP-RU by modulating NK cell activity [295], and increased MAIT cell infiltration has been shown to correlate with improved prognosis in hepatocellular carcinoma [296]. Furthermore, γδ T cells also have potent anti-tumour potential, with γδ T cell-deficient mice showing a significantly elevated incidence of tumours in murine models [297], [298], [299], [300]. In humans, intratumoural γδ T cells were the most favourable prognostic indicator across 39 malignancies [301], and indeed γδ T cell frequency is associated with overall or disease-free survival in melanoma patients [302], [303], [304] and leukemia patients receiving allogenic bone-marrow transplantation [305], [306]. *In vitro* studies suggest Vδ1+ T cells have higher anti-tumour cytotoxicity compared to their Vδ2+ counterparts [287], [307], [308], [309]. In renal cancer patients, Vδ1+ T cells positively correlated with tumour burden, while Vδ2+ T cells negatively correlated, suggesting these two subsets have different roles in this cancer setting [310] and aging may have a differential effect on this cancer as aging leads to the loss of the Vδ2+ T cell population and not the Vδ1+ T cells. The impact of aging on the anti-tumour role of unconventional T cells remains to be investigated, but given that there is an age-dependent reduction in human MAIT cells, Vδ9+Vδ2+ T cells, and possibly iNKT cells with age, and as reduced frequencies of MAIT cells, γδ T cells, and iNKT cells have been associated with poor survival in several cancers, the age-dependent decline of these cells may contribute to the higher incidence of cancer in older individuals and has important clinical implications.

On the other hand, unconventional T cells have also been shown to promote tumour development and are associated with poor survival in some settings. IL-17 is pro-tumourigenic in various cancers, promoting tumour growth, angiogenesis, and the induction of myeloid-derived suppressor cells [311]. γδ T cells, particularly Vδ1+ T cells, have been found to be a major source of IL-17 in various human cancers [312], [313] and murine tumour models [314], [315]. IL-17 and IL-22 production from NKT17 cells [316], [317] and IL-17 production from MAIT cells [318] have also been shown to contribute to tumour growth and metastasis, with a recent single cell analysis of tumour-infiltrating T cells across 21 cancer types showing that 50% of intratumoural Tc17 cells were MAIT cells [319]. Recently, it has been demonstrated that there is a skewing of murine γδ T cells and iNKT cells towards an IL-17-producing phenotype with increasing age in tissues and thus this bias may be a crucial contributor to the age-related increase in tumour incidence. Indeed, in aged mice, IL-17-producing γδ T cells dominated the γδ T cell population in peripheral lymph nodes and had a direct pro-tumorigenic role in this tissue [94]. IL-17-producing γδ T cells are increased in an age-dependent manner in the lungs [95], adipose tissue [97], [320], and peripheral lymph node [94], while hepatic iNKT cells from aged mice produce higher levels of IL-17 compared to young iNKT cells [166]. Although a direct role for IL-17 from aged iNKT cells in murine tumour models has not been demonstrated, HSV-2 or MCMV infection led to significantly higher levels of IL-17 in older mice, compared to younger mice, and was associated with increased neutrophil recruitment to the liver and chemokine production [166]. Notably, hepatic iNKT cells from aged mice produced higher levels of IL-17 compared to young iNKT cells, and adoptive transfer of aged iNKT cells into young mice resulted in hepatic injury [166], [167]. These reports may be relevant in tumour development as viral infections and chronic inflammation are known to trigger tumourigenesis. Thus IL-17 production from these unconventional T cells may contribute to the high incidence of cancer associated with old age. Of note, in some cases IL-17-production can have anti-tumour roles [321], [322], and increased IL-17-producing γδ T cells in aged mice provided protection from melanoma [95], thus the role of IL-17 in cancer in the elderly is likely heterogenous and tissue- and tumour-dependent. Given the emerging evidence in aged murine models, it will be important to establish whether this increase in IL-17 production from unconventional T cells with age occurs in humans, particularly in tissue-resident MAIT cells.

### 5.6 Role in tissue homeostasis/barrier immunity and the effect of aging

Aging is accompanied by a gradual increase in cell and tissue damage, as well as reduced tissue repair capabilities [323]. γδ T cells, in particular the Vδ1+ T cell subset, are abundant in the skin and promote wound healing by secreting keratinocyte growth factor (KGF) and insulin-like growth factor 1 (IGF1) [324], [325]. Interestingly, γδ-DETCs are found in equivalent numbers in young and aged murine skin, but following wounding, γδ-DETC numbers declined significantly in aged skin and delayed wound re-epithelization [326]. Similarly, iNKT cells have recently shown to play a role in orchestrating tissue repair in the skin [327] as well as in the liver [328]. In addition, the recent discovery that MAIT cells can be recruited to the wound and accelerate wound healing in the skin through amphiregulin production [189], [193], [201], [202], [203] suggests that these unconventional T cells together play a critical role in wound healing and protection of barrier integrity. Whether there is a decline in MAIT cell and iNKT cell tissue repair responses with age is an interesting area for future research. Crucially, αβ+ and Vδ1+ T cells isolated from chronic, non-healing wounds in the human skin have been found to be functionally impaired, with the inability to produce IGF1 and IL-2 upon stimulation [329]. Chronic wounds are a serious clinical problem common in the elderly and diabetic patients [329], and the reduced frequency or function of unconventional T cells in wound healing as suggested by these studies could contribute to this pathology.

Dysregulated gut homeostasis is a major driver of age-related inflammatory pathologies, with the loss of barrier integrity and gut dysbiosis leading to the leakage of bacteria and microbially derived products into circulation, which in turn contributes to inflammaging [10]. Unconventional T cells, with their role in host enteric defense and tissue repair function, play a vital role in gut homeostasis maintenance. Firstly, γδ T cells are abundant in the epithelium of the intestine and γδ IELs are critical for promoting epithelial integrity and healing [330], but γδ IELs have been shown to diminish in the gut of aged mice [101], [102], [103]. MAIT cells and γδ T cells in the lamina propria also protect gut integrity by producing IL-22, which promotes epithelial cell survival and antimicrobial peptide expression [331], and IL-17, which regulates occludin to prevent excessive barrier permeability during epithelial injury [332]. The loss of these cytokines from gut MAIT cells in Type 1 diabetes interestingly weakens barrier integrity [333]. A reduced frequency of these subsets in the elderly may therefore lead to leakage of microbial products from the lumen of the small intestine. Additionally, γδ IELs [334] and iNKT cells [335] have been shown to be important for the regulation of mucosal immunoglobin A (IgA) response, as well as intestinal T_regs_. Furthermore, unconventional T cells have been shown to modulate the composition of the commensal bacterial populations [336], [337]. Failure in these regulatory mechanisms leads to enhanced gut permeability and dysbiosis, and to what extent this may be attributed to the aging of unconventional T cells is still unknown. However, MAIT cells are one of the first immune cells exposed to gut microbes and their metabolites during dysbiosis, and this is thought to promote a cytotoxic and activated MAIT cell phenotype in the gut and liver [338], [339]. Indeed, MAIT cells have been shown to promote inflammation in high-fat diet-induced dysbiosis, leading to impaired glucose and lipid metabolism [340]. Furthermore, activation of MAIT cells by these microbes in the circulation may further contribute to the loss of MAIT cells in the periphery with age due to AICD [226], [341]. Thus, MAIT cell disruption with age may contribute to, and be accelerated by, a dysfunctional gut microbiota.

Aging is often accompanied by obesity, and inflammation in the VAT in obesity is a major driver of insulin resistance associated with the development of type 2 diabetes and other age-associated metabolic dysfunctions [342]. Adipose iNKT cells characteristically secrete IL-10 and IL-2, which is required for suppressive T_reg_ expansion and function in adipose tissue, but iNKT cells are reduced in obesity in humans and mice [343], [344]. Interestingly, an age-dependent increase in IL-17-producing PLZF+ γδ T cells has been reported in murine VAT of lean adolescent and young-adult mice (20–28 weeks), which inversely correlated with the frequencies of iNKT cells and type 2 innate lymphoid cells (ILC2) [97]. Il-17 from these γδ T cells promoted stromal cell production of IL-33 in VAT, which was critical for increasing adipose T_regs_ frequencies at around 20 weeks old, as well as for the regulation of thermogenesis following cold challenge [97]. However, in elderly mice (19–24 months), γδ T cells were found to accumulate with age independently of fat mass, and promoted chronic inflammation, with γδ T cell deficiency reducing systemic IL-6 levels and improving the metabolic phenotype [96]. A high-fat diet augmented this age-dependent increase of γδ T cells, which was specific to VAT and not observed in the skin, blood or spleen, and human γδ T cell frequencies were also found to correlate with age in VAT [96]. Thus, adipose tissue T_reg_ homeostasis is maintained by iNKT cells in young mice, then as γδ T cells expand at 20 weeks in lean, young adult mice, they take over this protective role [97], [343], while in obesity and in old age, γδ T cells may contribute towards inflammation in VAT [96], [345], [346]. Finally, both human and mice studies point towards a deleterious, proinflammatory role for MAIT cells in obesity and metabolic diseases, where they are depleted from blood and increase in frequency in the adipose tissue, with high IL-17 and GrB expression [340]. However the effect of age on the role of MAIT cells in adipose tissue remains to be investigated.

Finally, the accumulation of senescent cells with age contributes to the chronically inflamed inflammaging environment observed in old age, which in turn drive the progression of age-related diseases [347], [348]. Removal of these senescent cells depends on the perforin-granzyme pathway and has been shown to be mediated by NK cells [349], but activated iNKT cells were also recently shown remove senescent cells in vivo [350]. Specifically, activated iNKT cells were able to remove senescent preadipocytes that accumulated in the adipose tissue of mice fed with a chronic high-fat diet, and could reverse the fibrosis induced by lung injury by removing senescent cells [350]. Elimination of senescent cells by iNKT cells may therefore have a vital role in healthy aging, as these senescent cells compromise tissue homeostasis by secreting an array of growth factors, proinflammatory cytokines and proteases [348]. Whether age influences the ability of these cells to remove senescent cells is unknown, as has been shown in NK cells [348].

### 5.7 Role in neurological diseases and the aging brain

Aging is also associated with increased incidences of neurodegenerative diseases such as dementia and Alzheimer’s disease (AD). Interestingly, a recent study of MAIT cells in the brain barrier tissues showed that MAIT cells were present in the meninges and choroid plexus of in C57BL/6 mice and increased with age (up to 18 months) in both tissues [351]. There was an age-dependent cognitive decline in young adult MR1-/- mice which could be rescued by the adoptive transfer of MAIT cells, but not conventional CD4+ or CD8+ T cells, into MR1-/- mice [351]. This is similar to the meningeal γδ T cells which control synaptic plasticity and short-term memory through the secretion of IL-17 [352]. In contrast to this protective role of MAIT cells and γδ T cells for normal cognitive function, these cells promote neurodegeneration in AD models, possibly due to pathophysiological dysregulation [353]. Despite the pro-cognitive role of IL-17 in healthy meninges, IL-17 produced by meningeal γδ T cells promoted synaptic dysfunction in an AD model in mice [353]. MAIT cells also promoted AD development in mice, and AD patients have an increase in MR1 expression in the microglia surrounding plaques in the brain [354]. A TCR γ repertoire profiling study of AD patients also found that there is an age-related reduction in the number of clonotypes in the cerebral cortex, in both non-dementia donors and AD patients, with TRGV9 clonotypes decreasing and TRGV2/4/8 segments increasing with age, which was different to the blood [355], suggesting similar to some mouse tissues, there is a skewing of γ chain usage in the aging brain.

The incidence of stroke also increases with age, and γδ T cells are thought to have a pathogenic role in stroke through the production of IL-17 [356] which contributes to the infiltration of neutrophils and destruction of the blood-brain barrier [357], [358]. Antibiotic-induced intestinal dysbiosis affected stroke by inhibiting intestinal γδ17 T cells trafficking from the gut to the meninges [359]. Interestingly iNKT cells participate in protection against post-stroke bacterial infections [360], [361]. Thus, unconventional T cells have both protective and pathogenic roles in the brain, but more research is needed to determine whether the inflammatory conditions of aging may skew these cells towards a pathogenic role.

## 6 Perspectives and remaining questions

**Fig. 3:**
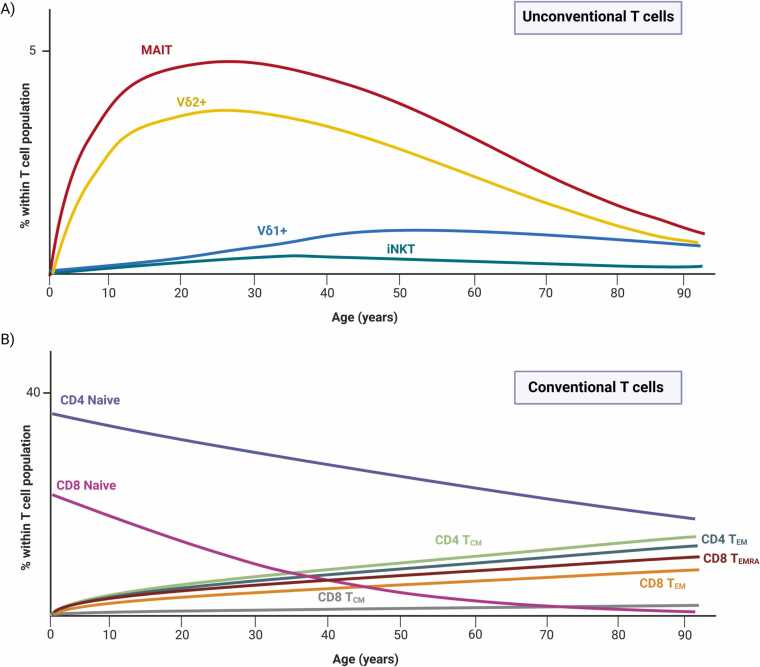
Aging of unconventional T cells in humans. A) Schematic of unconventional T cell frequencies within T cells over the human lifespan, based on current data. Within γδ T cells in cord blood, the Vδ1+ subset is the dominant γδ T cell subset but the Vδ2+ subset expands rapidly after birth, comprising most of the γδ T cell population by 1 years. MAIT cells also rapidly expand after birth, comprising most of the CD161++Vα7.2+ population by 1 years. MAIT cells and Vδ2+ populations increase to reach adult levels in early childhood, peaking around 25 years of age, and then decline with advancing age thereafter. iNKT cells remain relatively stable between birth and adulthood and may gradually decline over time depending on the cohort. Based on γδ T cell data from [77], [79] and literature reviews from [30], [392], [393], MAIT cell data from [11], [71], [213], iNKT cell data from [71], [144], [394]. Gender and geographic origin/race may have an effect on average frequencies, particularly in γδ T cells as the Vδ1+ population expands in CMV-seropositive donors [393], and MAIT cell frequencies are lower in non-western countries [215]. Age-dependent decline of MAIT cells and Vδ2+ cells is faster in males. B) Schematic of conventional CD4+ and CD8+ T cell memory subset frequencies within T cells over the human lifespan, shown for comparison. Memory subsets are defined by CCR7 and CD45RA expression, where naïve T cells are CCR7+CD45RA+, central memory T cells (T_CM_) are CCR7+CD45RA‐, effector memory T cells (T_EM_) are CCR7-CD45RA-, and terminally differentiated T cells (T_EMRA_) are CCR7‐CD45RA+. Naïve cells decrease with age while memory T cell subsets increase with age, with the CD8+ T cells in particular becoming dominated by memory subsets in the elderly. Conventional T cell data is based on [395], [396]. Created with Biorender.com.

While there are many outstanding questions that remain, current review of the literature highlights some interesting similarities in the effect of aging on unconventional T cell populations. In humans, following expansion in early life, MAIT cells and Vδ2+ T cells peak around 25 years of age and decline with increasing age thereafter (Fig. 3), both in frequency and absolute numbers. iNKT cell frequencies may also decline depending on the cohort. In contrary, Vδ1+ T cells are maintained or increased with increasing age. The rise and fall of MAIT cells and Vδ2+ T cells with age in the periphery mirror each other and suggests similar factors may drive this phenomenon. Vδ2+ T cells mainly consist of Vγ9+Vδ2+ T cells, which have been found to be the more “innate-like” subset within Vδ2+ T cells, compared to Vγ9‐Vδ2+ T cells [23], and contain the CD26++ subset that transcriptionally have been found to be more MAIT-like [24]. In contrast, Vδ1+ are tissue-resident and more adaptive [15]. Thus, there is a dichotomy within unconventional T cells, as the more “innate-like”, circulating subsets – MAIT, Vγ9+Vδ2+, and possibly iNKT cells – are reduced in old age, whereas the more adaptive, tissue-resident subset – Vδ1+ cells – are maintained in the elderly. This suggests a common mechanism affecting the “innate-like" members of the unconventional T cell family in the elderly.

The factors driving the loss of Vγ9+Vδ2+ T cells and MAIT in the human elderly is still unclear. They may be recruited to the tissues in response to inflammation or infection, as clearly observed for MAIT cells and Vγ9+Vδ2+ T cells in COVID-19 [270], [362]. Additionally, as Vγ9+Vδ2+ T cells and MAIT cells undergo clonal expansion very early in life and are continuously stimulated throughout life, they may be replicatively senescent. MAIT cells indeed have shorter telomere lengths compared to conventional memory CD8+ T cells and are thought to be hypoproliferative in adults [179], [363]. However, both Vδ2+ and Vδ1+ T cells have equally reduced telomere lengths in middle age and older adults [364], suggesting they are both replicatively senescent, and that other mechanisms may explain the different effects of age on these subsets. A more likely explanation is that the loss of Vγ9+Vδ2+ T cells and MAIT cells may be due to increased sensitivity to AICD in these cells [77], [80], [214]. It is particularly interesting that the frequency of MAIT cells expressing the apoptosis marker Annexin V correlated with age, as well as MAIT cells expressing the activation marker CD69 [214]. “Innate-like” T cells have high expression of cytokine-receptors and thus the inflammaging environment, combined with microbial products from age-induced dysbiosis, may lead to overactivation of these cells. Intriguingly, inflammaging cytokines IL-1β, IL-6, TNFα and IL-18 are all found at significantly higher levels in men compared to women [365], [366], [367], although they increase with age in both genders [367], [368]. Both MAIT cells and Vδ2+ T cell reduction in the elderly has been reported to be faster in men compared to women, whereas in iNKT cells there have also been conflicting reports of age-dependent decline specifically in women [153] or men [152]. Serum IL-18 levels have previously been shown to negatively correlate with MAIT cell frequencies in various inflammatory diseases such as in multiple sclerosis [369], and correlates with MAIT cell activation in fatal cases of COVID-19 [269]. Thus, it is possible that increased AICD of Vδ2+ T cells and MAIT cells in the elderly is accelerated by inflammaging cytokines that are higher in men compared to women. It would be interesting to see if the reduction of inflammaging with anti-cytokine (IL-1β, IL-6, IL-18 and TNFα) antibodies may help restore unconventional T cell numbers in the elderly.

Additionally, the lack of homeostatic cytokines maintaining these populations in the periphery may contribute to the loss of these cells in old age. In mice, IL-15 has been shown to be critical for the survival and homeostatic proliferation of memory T cells and NK cells [370], [371], as well as NKT1/NKT2 subsets of iNKT cells [371], [372], [373], and γδ T cells, in particular γδ DETCs and γδ IELs cells [371], [374]. In humans, IL-15 preferentially induces the expansion of the CD4- iNKT cell subset, while IL-7 induces the proliferation of the CD4+ iNKT cells [375]. IL-15 also induces significant proliferation of human MAIT cells, but not IL-7 [376]. Interestingly, it has been shown that conventional memory CD8+ T cells and NK cells compete with iNKT cells for IL-15 and their expansion can be limited in vivo by other IL-15-responsive populations in mice [372]. Given that both memory CD8+ T cell (Fig. 3) and NK cell numbers increase in old age [10], [377], it will be intriguing to test whether competition for IL-15 may contribute in part to the reduced frequency of these IL-15-responsive unconventional T cell populations. Furthermore, NKT17 cells are dependent on IL-7, not IL-15, for their survival [373], while IL-15R signaling inhibits, and IL-7 enhances, the expansion of γδ17 cells [94], [374], suggesting that subset-specific differences in homeostatic cytokine requirements may affect the skewing of these cells towards the type-17 phenotype. In support of this hypothesis, increased IL-7 in the lymph node of old mice expanded pro-tumourgenic γδ17 cells [94]. T_reg_ cells can also suppress the proliferation of iNKT cells [378], so the dramatic increase in T_regs_ with age – also driven by IL-15 [379] – may play a part in the reduction in iNKT cells, and potentially other unconventional T cells. Other cytokines such as IL-21 in iNKT cells [380] and IL-23 and IL-18 in MAIT cells [209], [380] are also known to be important for their homeostasis, so the effect of age on the homeostatic maintenance of these cells in the elderly requires further investigation. If the lack of homeostatic cytokines maintaining unconventional T cells does indeed contribute to their decline in old age, perhaps due to competition with oligoclonal expansions of conventional T cells, restoring their numbers by supplementing with cytokines may be an interesting avenue of research.

**Fig. 4:**
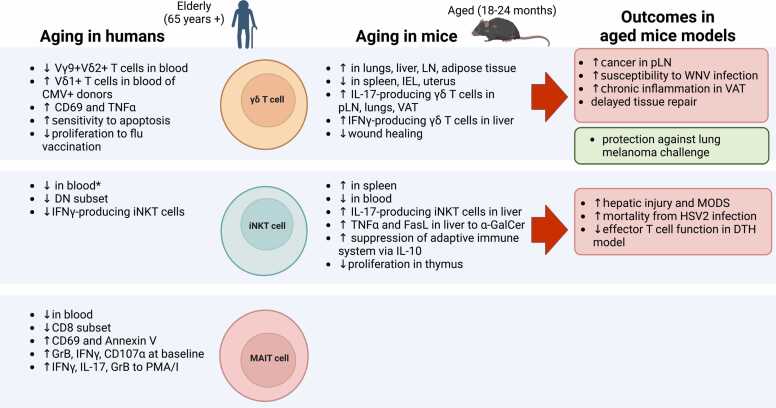
Effect of aging on unconventional T cells in humans and mice. Schematic of the effect of aging in humans and mice on unconventional T cells, based on current data. The effects of aging on γδ T cells, iNKT cells and MAIT cells are summarised, with findings from human studies on the left and murine studies on the right. The consequences of these effects as demonstrated in mouse models referred to in the text are highlighted in red or green boxes, with red showing where they have been shown to play a pathogenic role and green showing a protective role. In general, there is consistent increase in IL-17-producing γδ T cells and iNKT cells in certain tissues in aged mice, and a reduction in Vγ9+Vδ2+ T cells and MAIT cells in human blood. *The reduction of iNKT cells in human blood with age is cohort dependent. The effect of aging in MAIT cells in mice and consequences of these effects in humans remains to be explored. LN=lymph node, pLN=peripheral lymph node, IEL=intraepithelial lymphocytes, WNV= West Nile Virus, HSV2 =herpes simplex virus 2, PMA/I = PMA/ionomycin, VAT=visceral adipose tissue, MODS=multi-organ dysfunction syndrome. Created with Biorender.com.

In mice, where most studies are on tissue-resident cells, there is a clear age-dependent increase of γδ T cells and iNKT cells producing IL-17 in certain tissues (Fig. 4). Increased IL-17 from γδ T cells in aged mice increased tumour development in peripheral lymph nodes [94] and chronic inflammation in adipose tissues [96], while increased IL-17 from aged iNKT cells led to increased hepatic injury and mortality following HSV-2 infection [166]. Age-skewed responses can also be protective in a tissue-dependent manner, as demonstrated for IL-17 from aged γδT cells in a lung melanoma challenge model [95]. Whether murine MAIT cells may also have increased IL-17 production in aged tissues requires further investigation. Augmented responses have been also reported in human blood MAIT cells from the elderly, with increased activation [11] and production of IFNγ and IL-17 [214], as well as in peripheral γδ T cells, with increased CD69 expression, TNFα production, and proliferation to flu vaccination [67], [282]. However, increased basal activation may impair protective functions of MAIT cells and Vδ2+ cells, as both subsets may be more sensitive to apoptosis [69], [75], [214], and hampered specific MAIT cell responses to *E. coli* and influenza in vitro [11]. Furthermore, the reduction in absolute numbers of MAIT cells and Vγ9+Vδ2+ T cells in the elderly may lead to lower overall effector cell numbers [282]. Further research into whether this activated phenotype is found in human tissues will have important clinical implications, as they will likely have both protective and pathogenic consequences depending on the tissue, as seen in mice.

Studying unconventional T cells in the context of age is particularly challenging due to subset-specific differences between human and mice biology. γδ T cells express different γ and δ chains in mice and humans. MAIT cells are abundant in humans but rare in mice [178], [188], posing challenges in confirming their functions particularly in tissues, whereas the opposite is true for iNKT cells, making it difficult to translate findings in mice into humans. Recent research also highlights the ability of all three of these populations to partially compensate for each other in knock-out models. For example, MAIT cells are increased in iNKT cell-deficient *CD1d-/-* mice [209], as well as in γδ T cell-deficient *Tcrd-/-* mice [202], [210], [381], although these cell types have unique antigenic targets so they are unlikely to be redundant and point towards competition for shared factors. The high inter-donor variability in the frequency of these unconventional T cells in healthy humans also means that to study the effect of aging on these cells requires large cohorts with sex-, BMI-, and age-matched controls. This may make it particularly difficult to thoroughly investigate what happens to these cells in aging tissues in humans, of which there is very little information of. Thus, animal studies will be key for us to increase our understanding of the function and role of these cells in old age. However, it is important to emphasize that while humans experience several infections during their lifetime, laboratory mice are kept in a sterile environment their entire life. Given the importance of microbial exposure to the expansion, function, and tissue distribution of unconventional T cells [12], the effect of aging on these T cells may differ between mice and humans. Indeed, the functional distinction between type-1, type-2 and type-17 unconventional T cells are hard-wired in the thymus in mice [47], [210], whereas in humans these functional subtypes are interchangeable and plastic [382].

Further investigations are required to define what happens to these unconventional T cells in the elderly, particularly in terms of their senescence and exhaustion. CMV is a major driver of conventional T cell immunosenescence due to chronic stimulation of CD8+ T cells, inducing clonal expansions, accelerated terminal differentiation and increased replicative senescence [383]. Relatively little is known about the effect of CMV on unconventional T cells aging, but Vδ2- T cells are increased CMV-seropositive donors compared to CMV-seronegative donors in all age groups, and this difference is the most significant in older age groups [384]. These cells are terminally differentiated T_EMRA_ γδ T cells, similar to CMV-specific αβ T cells [383], [385], [386]. A recent study of 1000 donors also reported lower numbers of iNKT cells (as defined by Vα24+CD161++ T cells) and MAIT cells in CMV-seropositive donors [387], but further research is required to understand whether CMV influences their differentiation and function. It is also unknown whether unconventional T cells show signs of exhaustion in the elderly, which is also relevant given that there is increasing interest in the role of unconventional T cells in immunotherapies including checkpoint blockade therapy [388], chimeric antigen receptor (CAR) T cells [389], and vaccines [362], the effectiveness of each which may be modulated with age. Single-cell RNA-seq studies will help elucidate the heterogeneity in these populations with age that may have been masked in bulk gene expression studies or flow cytometry experiments [98]. Additionally, there is a lack of aged studies into MAIT cells compared to iNKT cells and γδ T cells, but the recent development of MR1 tetramers [178] should allow their examination in vivo where they are rare. Furthermore, dysregulated and chronic activation of unconventional T cells can lead to increased IL-17 production as observed in many autoimmune conditions and cancers, and whether these cells negatively contribute to the inflammaging environment remains to be determined. Studying the genetic and epigenetic landscape of unconventional T cells in the young and old will uncover changes that will shed more light on the effects of aging on these cells. Ultimately, these findings will enable the therapeutic intervention of unconventional T cell aging.

## 7 Conclusion

Unconventional T cells are emerging as critical players in all aspects of immunity, including infection, autoimmunity, cancer, tissue homeostasis, metabolic diseases, neurological diseases, and vaccine responses. While we are only beginning to understand how these cells age and contribute to age-associated diseases, there are striking similarities in the changes observed with aging between “innate-like” unconventional T cell subsets which may have implications for their shared biology. Recent evidence supports the idea that unconventional T cells compete for niches within tissues and may have overlapping functions, as expertly reviewed by [12], [47]. Indeed, MAIT cells are expanded in the thymus, spleen and liver of CD1d knockout mice lacking iNKT cells [209], and this idea was also recently confirmed in a patient with a homozygous point mutation in MR1 associated with an absence of MAIT cells, but an expanded Vδ9+Vδ2+ T cell population [390]. The combined loss of both MAIT cells and Vδ9+Vδ2+ T cell in the elderly would remove this niche, leaving these individuals highly susceptible to various bacterial and viral infections, as well as age-related pathologies. In light of these findings, studying these unconventional T cells together and how aging affects this functional niche will illuminate therapeutic strategies that will be relevant in a wide range of age-associated diseases.
